# Sex Determination of the Live Rubber Plantation Litter Beetle, *Luprops tristis*: A Novel Method

**DOI:** 10.1673/031.008.1201

**Published:** 2008-02-20

**Authors:** K.V. Vinod, Thomas K. Sabu, T. M. Benny

**Affiliations:** Litter Entomology Research Unit, P.O. & Research Department of Zoology, St. Joseph's College, Devagiri, Calicut, Kerala, India 673008

**Keywords:** Coleoptera, Tenebrionidae, *Mupli beetle*, sternal notch

## Abstract

Absence of a discrete, externally visible gender-specific character makes sex determination of the rubber plantation litter beetle, *Luprops tristis* Fabricius (Coleoptera: Tenebrionidae: Lupropini), a difficult task. A new method based on a distinct notch on the 8^th^ sternite of males that can be used to distinguish the sexes is described. This is the only method by which accurate sex determination of *L. tristis* could be done when culturing of live specimens is required. All alternative methods were found to be either inaccurate or led to higher mortality.

## Introduction

**Figure 1.**
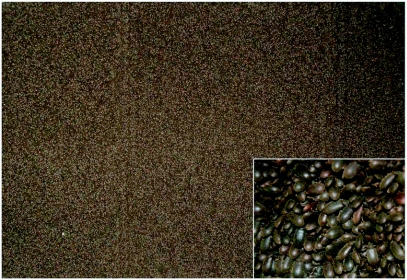
Aggregated, dormant beetles settled on the wall of a residential building.

Seasonal mass invasion of huge aggregations of a litter dwelling beetle, *Luprops tristis* (Fabricius 1801) (Coleoptera: Tenebrionidae: Lupropini), locally called as ‘*Mupli beetle*’ numbering *ca* 0.5–4 million on residential buildings following summer showers is a regular event in the rubber tracts along the western slopes of Southern Western Ghats. Continued presence of clusters of thousands of nocturnally active beetles crawling inside the house is a frustrating nuisance of unimaginable magnitude (Figure 1). Though the beetles do not sting or bite, when disturbed by picking them off the walls or when they are squashed or pressed after falling from the ceiling, they release an irritating, odoriferous phenolic secretion leading to blisters. The amazingly synchronous mass invasion, abundance and selection of specific buildings as shelter that has been observed for more than 20 years, remains a curious ecological phenomenon to biologists, and one of great agony and concern to the affected people in rubber plantation belts. Despite three decades of their widespread presence in the region, efficient strategies for controlling the population build up of *L. tristis* have not been developed. There is a critical need to develop methods of *L. tristis* beetle suppression.

Recent studies of the biology and dormant period of *L. tristis* (Sabu et al. 2007) indicated that further studies by maintaining lab cultures are essential to 1) analyze how variation in age affects the fecundity and fitness of beetles after dormancy, 2) determine the optimum and minimum duration of feeding time required during predormancy to carry the organism through dormancy, 3) determine the environmental regulators of dormancy and the hormonal systems that direct the onset and termination of dormancy and 4) elucidate the reproductive physiology prior to and after dormancy. Further, their astonishing abundance and ease of rearing in laboratory conditions makes *L. tristis* an excellent model for advanced studies of dormancy in tropical insects including the molecular biology and identification of genes implicated in dormancy (Denlinger 2002). Nevertheless, absence of externally visible sexual dimorphic characters makes sex determination of adults a difficult task, a situation common among tenebrionids (Crowson 1981). A safe and easy method for sex determination of adults is needed for these studies.

There are two methods commonly used for sex determination of tenebrionids (Bhattacharya and Waldbauer 1970; Lawrence et al. 1999), as well as a new method for *L. curticollis* (Thomas and Jacob 2005). In many Tenebrionidae, the anterior edge of sternites 8 and 9 in males are without a median strut (Lawrence et al. 1999). In females of *Tenebrio molitor*, there is little or no separation between the 3^rd^ , 4^th^ and 5^th^ sternites, while in males the intersegmental membranes are clearly visible with a lighter coloring. Moreover, the 5^th^ visible sternite is slightly pointed in the female and quite round in the male (Bhattacharya et al. 1970). However the total absence of all such characters in *L. tristis* leaves no scope for employing them to differentiate the sexes. Size ranges widely overlap in male and females of *L. tristis*, which makes gender based size variation (Thomas and Jacob 2005) not useful.

**Figure 2.**
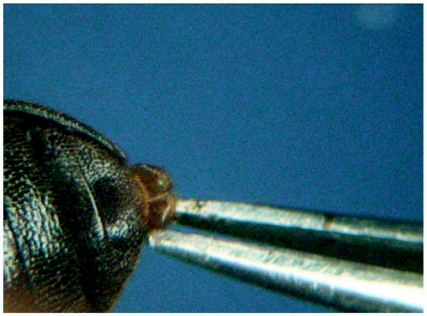
*Luprops tristis* being sexed by pulling out the 8^th^ sternite with forceps.

While searching for alternate methods, the partial extrusion of genitalia by squeezing the abdomen for sex determination (Agee 1964) is possible in *L. tristis*. Further, a previously unrecorded sexual dimorphic character in the 8^th^ sternite was detected. In the current work, a new method, which we call the ‘sternal notch method,’ is described and compared with other methods for sex determination of *L. tristis*.

## Materials and Methods

Newly emerged (teneral) adults of *L. tristis* were collected from a heavily infested rubber plantation at Thamarassery, 30 km away from Calicut city, by the litter sifting method in the first week of March 2005. Teneral adults can be easily distinguished by the light brown colouration of the body, which lasts for 5–6 h after eclosion (Sabu et al. 2007). 500 beetles were collected, kept in four (2 × 2 mm) large clay vessels (13 × 35 cm) half filled with soil and freshly fallen rubber litter and covered with nylon net. Field collected fallen leaves of varying ages were provided as food. A circle of filter paper moistened with drops of distilled water was placed in a petri dish, which was kept in each clay vessel. One hundred beetles each were sexed by the abdomen squeezing method and by male-female size variation. Two hundred beetles were sexed by the sternal notch method described below. Two-day old beetles were used in all tests.

The sternal notch method is based on the observation of a distinct notch in the 8^th^ sternite of males that is absent in females. When sexed by the sternal notch method, the beetle was held by the left thumb and index finger, placed on the stage of a stereo zoom microscope (25 X magnification) with the ventral surface facing up and with the posterior part of the beetle away from the worker, while the right hand was used to operate a # 4 watch makers forceps with blunted tips. Care must be taken to immobilize the hind legs, otherwise the beetle may rupture the defensive gland reservoirs with its legs and the oily defensive secretion will make identification difficult. Holding the beetles using a small piece of filter paper will solve this problem, but that may slow the operation. When the apex of the last visible abdominal sternite (7^th^) was gently lifted with the forceps, the edges of 8^th^ sternite and the last outer tergite (8^th^) became visible. Normally beetles extruded the tip of the 8^th^ sternite along with the tergite. Rarely, when beetle did not extrude the sternite, the tip of the sternite was gently pulled out until the sternite became completely visible (Figure 2). Care was given not to press the beetle hard or to pull the sternite out too far, or to rupture the defensive gland reservoirs. Beetles with and without a notch in the outer margin of the 8^th^ sternite, were segregated into designated vials (4.5 × 7 cm) and covered with nylon net (2 mm mesh size) with a 2 cm slit at the centre. Time taken for differentiating the beetles based on the sternal notch method was analysed by providing 100 beetles each to a postgraduate student with no previous experience and to an experienced lab assistant. Genitalia of 100 specimens sexed by the sternal notch method were squeezed out to verify the accuracy of the method. The rest of the specimens (N=100) sexed by the sternal notch method and those sexed by squeezing the abdomen (N=100) were cultured in separate clay pots till they entered in to dormancy (for one month). Mortality was recorded at weekly intervals.

**Figure 3.**
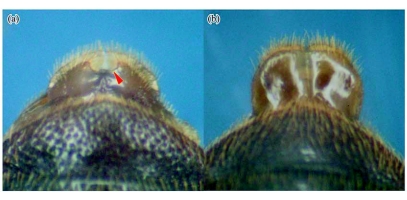
Posterior margin of 8^th^ sternite in *Luprops tristis* with semicircular median notch in male (a) and without semicircular notch in female (b).

One hundred beetles, cooled on ice (Sappington and Spurgeon 2000), were measured under a stereo zoom microscope with an ocular micrometer at 20 X magnification. Body length and width, (length = tip of the head to tip of the closed elytra; width = most expanded region near apex of the elytra) were measured. Sex of each beetle was confirmed by squeezing the genitalia out after the completion of measurements.

## Statistical analysis

Significance level of gender based body length variations was determined by a t-test. Gender-based variations in the mortality of specimens sexed by squeezing the abdomen was estimated with a Chi-square test (α = 0.05) (Zar 2003). Gretl open source software for Windows was used in statistical analyses.

## Results and Discussion

The new methodology is based on the presence of the distinct appearance of the outer border of the 8^th^ sternite of male and female *L. tristis*. In males, the 8^th^ sternite is semicircular with a distinct median semicircular notch in the outer border (Figure 3). The margin of the notch is setaceous, and setae are longer at its angles where the outer border of the sternite continues. The 8^th^ sternite in females is rectangular and larger, without the posterior-median semicircular notch and with indistinct outer angles.

An experienced person could sort 100 beetles in half an hour, while for the novice 2 hours were needed. The most common problem while learning the sternal notch method was that excessive pulling damages the sternite.

**Figure 4.**
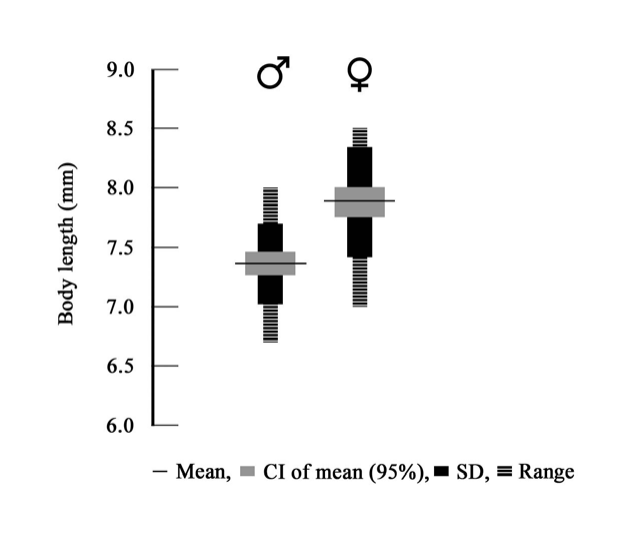
Gender based variation in body length of *Luprops tristis*. 43 males and 57 females were used for analysis. Differences in mean, 95% confidence interval (CI) of means, standard deviations and ranges between sexes are shown.

Gender-based difference in body length was significant (d.f. = 97, t = -6.48, *P* = 0.00), with females being larger than males. But the presence of larger males (> 7mm) and smaller females (< 8mm) in the population (Figure 4) makes it an unreliable method. With broad overlapping of ranges (7–8 mm; 88.4% males and 66.7% females) one is not sure whether sexing was correct, which may necessitate re-sexing by an alternate criteria requiring additional time and labor.

All beetles sexed by the ‘sternal notch method’ survived. Verification of the method by observation of the genitalia proved that the method was 100% accurate. In contrast, 43% of the beetles sexed by squeezing the abdomen died with in 1–4 weeks. Variation in mortality between sexes was significant (χ^2^ = 14.8, *P* = 0.00), with higher mortality in males (65.9%) than in females (27.1%). The higher mortality in males is likely due to mechanical damage arising from the squeezing out of genetalia, as partially extruded genitalia of 40% of male *L. tristis* were not fully withdrawn. In most cases (85%), the first abdominal ventral segment was separated from the metathroax. Our results are in conformity with the observations of Sappington and Spurgeon (2000) that squeezing out the genetalia of boll weevils is useful for sex determination only when culturing of live specimens is not required.

These results show that squeezing out the genetalia and gender based size differences are not useful methods for sex determination of *L. tristis* when culturing of live specimens is required. The sternal notch method was found to be a harmless, quick and accurate method for sexing adult *L. tristis* beetles. It is possible that this character would be useful for sexing other species of *Luprops* and genera of the tribe Lupropinii, and perhaps other tenebrionids.
